# Production of highly immunogenic and safe Triton X-100 produced bacterial ghost vaccine against *Shigella flexneri* 2b serotype

**DOI:** 10.1186/s13099-023-00568-7

**Published:** 2023-09-07

**Authors:** Amany Abdelfattah, Reham Samir, Heba M. Amin

**Affiliations:** 1 Department of Microbiology and Immunology, Faculty of Pharmacy, October University for Modern Sciences and Arts (MSA), 26 July Mehwar Road Intersection With Wahat Road, 6Th of October, 12451 Giza Egypt; 2 Department of Microbiology and Immunology, Faculty of Pharmacy, Cairo University, Nile Corniche, El Sayeda Zeinab, Cairo, 11562 Egypt

**Keywords:** *Shigella flexneri*, Triton X-100, Bacterial ghost vaccine, Shigellosis, Immune response, Bactericidal activity, Histopathology analysis

## Abstract

**Background:**

Bacterial ghost cells (BGCs) are cells were drained of their genetic and cytoplasmic components. This work aimed to develop vaccine candidates against the *Shigella flexneri* (*S. flexneri*) 2b serotype using the BGCs approach. For the first time, (*S. flexneri*) 2b serotype BGCs vaccine was prepared by incubation with Triton X-100 (TX100) for only 12 h. Its safety and immunogenicity were compared to another vaccine produced using a previously used surfactant, namely Tween 80 (TW80). Scanning electron microscopy (SEM), cellular DNA, protein contents measurements, and ghost cell re-cultivation were used to confirm the successful generation of the BGCs. Immunogenicity was assessed through mice's intraperitoneal (IP) immunization followed by infection with *S. flexneri* ATCC 12022. Finally, histopathological examination was carried out.

**Results:**

Viable colony forming units (CFUs) of *S. flexneri* were counted from stool samples as well as homogenized colon tissues of the non-immunized challenged group. Immunized mice sera showed a significant increase in serum bactericidal activity of both preparations (TX100 = 40% and TW80 = 56%) compared to the non-immunized challenged group (positive control). The IgG levels of the bacterial ghost-vaccinated groups were four and three times greater for the TX100 and TW80 ghost vaccines, respectively, compared to that of the positive control; both bacterial ghost vaccines (BGVs) were safe and effective, according to the results of the safety check tests and histopathological analysis.

**Conclusions:**

When comparing the BGVs prepared using TX100 and TW80 methods, the use of TX100 as a new chemical treating agent for BGC production attained robust results in terms of shorter incubation time with the targeted cells and a strong immune response against *S. flexneri* 2b serotype ATCC 12022 in the IP challenge test. However, a clinical study is needed to confirm the efficacy and total safety of this novel vaccine.

**Supplementary Information:**

The online version contains supplementary material available at 10.1186/s13099-023-00568-7.

## Background

*Shigella flexneri (S. flexneri)* is one of the four *Shigella* species and a non-lactose fermenter member of the *Enterobacteriaceae* family. *Shigella* species are Gram-negative bacilli, non-motile, and facultative anaerobes [1]. Based on biochemical variations and O-antigen variants, these species are further split into different serotypes. According to this classification, *S. flexneri* is divided into 13 serotypes [2]. *Shigella sonnei* (*S.sonnei*) and *S. flexneri* are the major species that cause the endemic sickness known as shigellosis; they infiltrate and colonize the intestinal mucosa, causing a series of clinical symptoms [3]. Shigellosis is characterized by fever, stomach pain, and bloody diarrhea, as well as the presence of erythrocytes, polymorphonuclear neutrophils (PMNs), and mucus in the patient's faeces [4]. Severe anorexia, lengthy diarrhea, malnutrition, weight loss, convulsions, large intestine dilatation, hemolytic-uremic syndrome, and kidney damage can all result from a complicated case of *S. flexneri*. Additionally, bacteremia can strike immunocompromised adults as well as newborns [5]. According to estimates, *S. flexneri* causes (60%) of shigellosis infections in low-income countries and has a higher death rate than other *Shigella* species [6, 7]. Many trials have been conducted over the years to create safe and effective *Shigella* vaccines, ranging from killed, live attenuated, and subunit vaccines [8].

Bacterial ghost cells (BGCs) are vacant bacterial cell envelopes that contain the majority of the antigenic markers found in active cells [9]. These ghosts serve as potent candidate vaccines, effective adjuvants, and efficient delivery mechanisms for RNA or DNA vaccines [10]. The concept of creating BGCs is based on creating holes and leaving them as empty shells, with complete evacuation of genetic and cytoplasmic contents [11]. Successful BGCs as vaccine candidates should preserve cell structural integrity while producing an efficient and safe immune response [12]. The E-lysis gene was the first technique for manufacturing BGCs, however, it showed several limitations, including a restriction to only Gram-negative bacteria and a high cost of manufacture [13, 14]. Another way of ghost preparation is the sponge-like technique, which is based on obtaining minimum inhibitory concentrations (MIC) of chemical agents that are capable of generating microscopic holes in the cell wall of bacteria, such as NaOH, SDS, CaCO_3_, and H_2_O_2_ [15]. That strategy paved the way for the generation of Gram-positive BGCs; however, deploying such a reagent was harmful to the targeted cells [16, 17]. Gram-negative *Salmonella enterica-serovar Typhimurium* BGCs were produced by employing a new chemical treatment by including Tween 80 (TW80) (7%). That technique was successful since it preserved the structural integrity of treated cells while producing an effective and safe immunological response [18, 19]. In our investigation, we’ve created two versions of *S. flexneri* BGCs. Triton X-100 (TX100) was used as a novel chemical treating agent to produce the first batch of BGCs. Its safety and effectiveness were compared to another batch of BGCs prepared with (TW80). In an in vivo mouse model of immunization followed by a challenge experiment, the humoral immune responses of the generated bacterial ghost vaccines (BGVs) were assessed. Finally, the effect of these vaccines on various tissues was studied using histopathology examination. This is the first study, to our knowledge, that characterize and assess the immunogenicity of the *S. flexneri* bacterial ghost vaccine (*Sf*-BGV) produced by (TX100).

## Results

### Minimum inhibitory concentration (MIC) determination of “TX100” against ATCC 12022

TX100 had a MIC of (6%) when tested against the ATCC 12022 strain. *Sf*-BGCs were produced using (5%) of v/v TX100 (sub-MIC value), Additional file 1: Table S1.

### Bacterial ghost cell (BGCs) production

Intact ATCC 12022 were successfully produced upon incubating 100 µL of *S. flexneri* 2b serotype ATCC 12022 (1 × 10^10^ CFU/mL) with (5%) v/v TX100/LB for 12 h**,** and (7%) v/v TW80/LB for 24 h. The pH was then adjusted to 2.8 by adding 180 µL of lactic acid and incubating for 1 h, Additional file 1: Tables S2, S3.

### Quality check for the produced BGCs

#### In vitro quality check

##### Light microscopic examination

Gram staining of *Sf-*BGC/TX100 and *Sf*-BGC/TW80 revealed Gram-negative bacilli, both of which retained their intactness and cellular integrity, Additional file 1: Tables S2, S3.

##### BGCs re-cultivation

After 24–48 h at 37 °C of cultivation of *Sf*-BGC/TX100 or *Sf*-BGC/TW80 pellets in LB broth and in DCA, no discernible bacterial growth was seen*,* Additional file 1: Tables S2, S3.

##### Released protein and DNA quantification

**Fig. 1:**
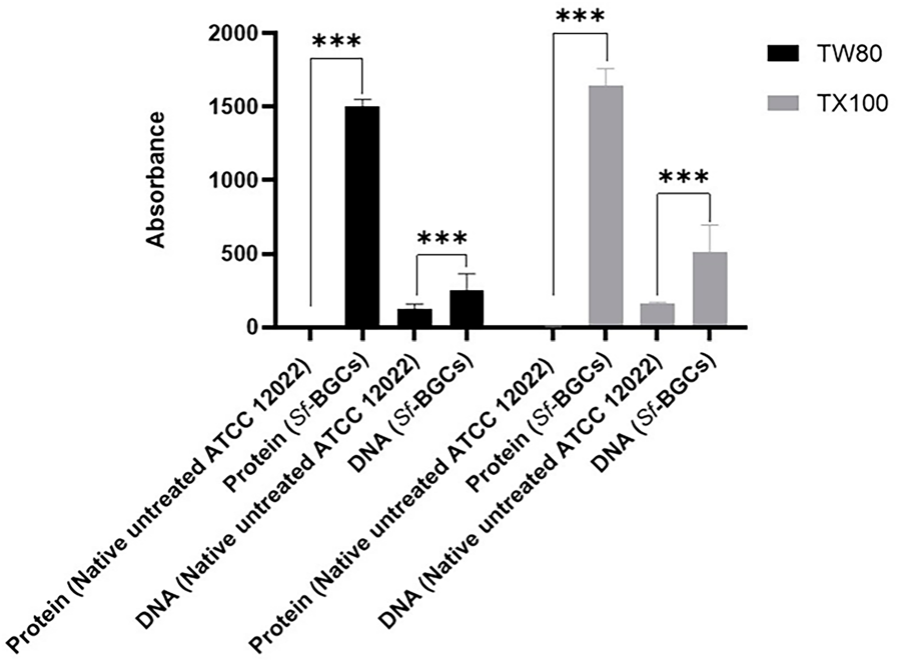
DNA and protein released in the culture supernatants of treated and untreated *S. flexneri* cells. A bar chart presenting the amount of released proteins and DNA from *Sf*-BGC/TW80 and *Sf*-BGC/TX100 compared to native, untreated cells (*S. flexneri* 2b serotype ATCC 12022) The X-axis represents the type of supernatant used in this experiment. The Y-axis represents the recorded absorbance of each preparation using Nanodrop at 280 nm and 260 nm to determine the amount of released proteins and DNA, respectively. (***) indicate a significant difference between the released amounts of protein and DNA, respectively, compared to native, untreated *S. flexneri* 2b serotype ATCC 12022. Data represent mean standard errors of the mean (SEM) obtained using one-way ANOVA data analysis. The difference is considered significant if P < 0.0001

When compared to the native, untreated cells of ATCC 12022 cultured for 12 h, *Sf-*BGC/TX100 showed a significant increase in the released DNA and protein of (68%) and (100%), respectively. On the other hand, *Sf*-BGC/TW80 demonstrated significant increases in the released DNA and protein of (50%) and (100%), respectively, compared to native untreated ATCC 12022 cells cultured for 24 h (P value < 0.0001 ANOVA analysis), Fig. 1.

##### Scanning electron microscopy “SEM” examination of “Sf- BGCs”

**Fig. 2:**
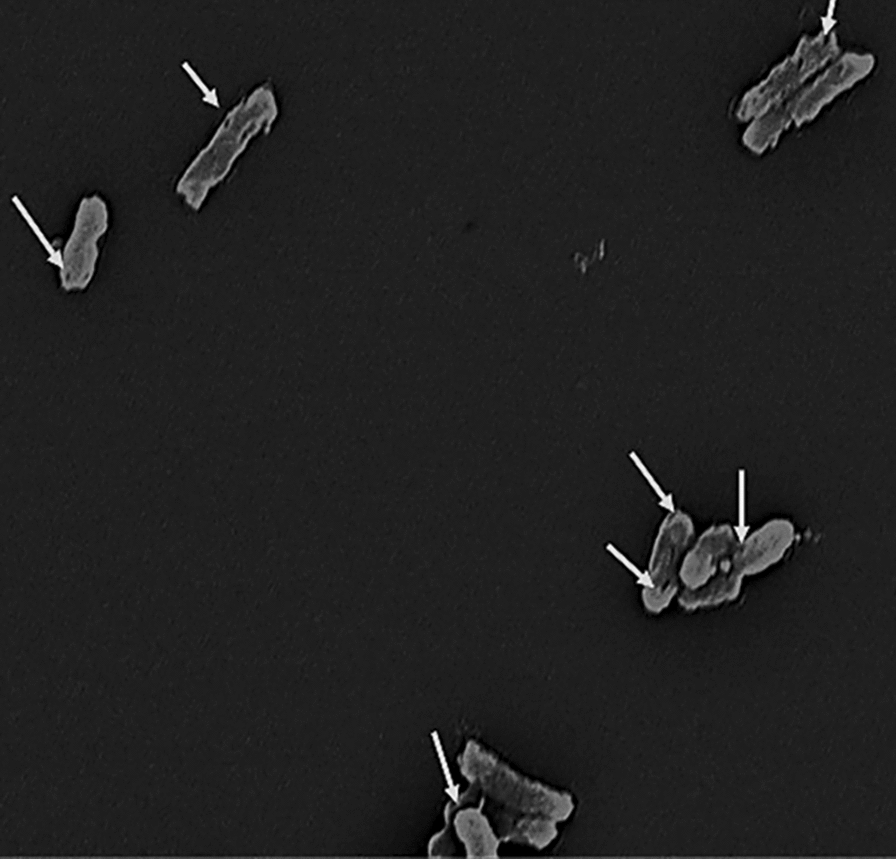
SEM image of *Sf*-BGCs/TW80. Scanning electron microscope (SEM) image of *Sf*-BGCs/TW80 pellet at magnification power × 16,000. White arrows point to a transmembrane tunnel formed in the cell walls of cells treated with (7%) v/v TW80 for 24 h

**Fig. 3:**
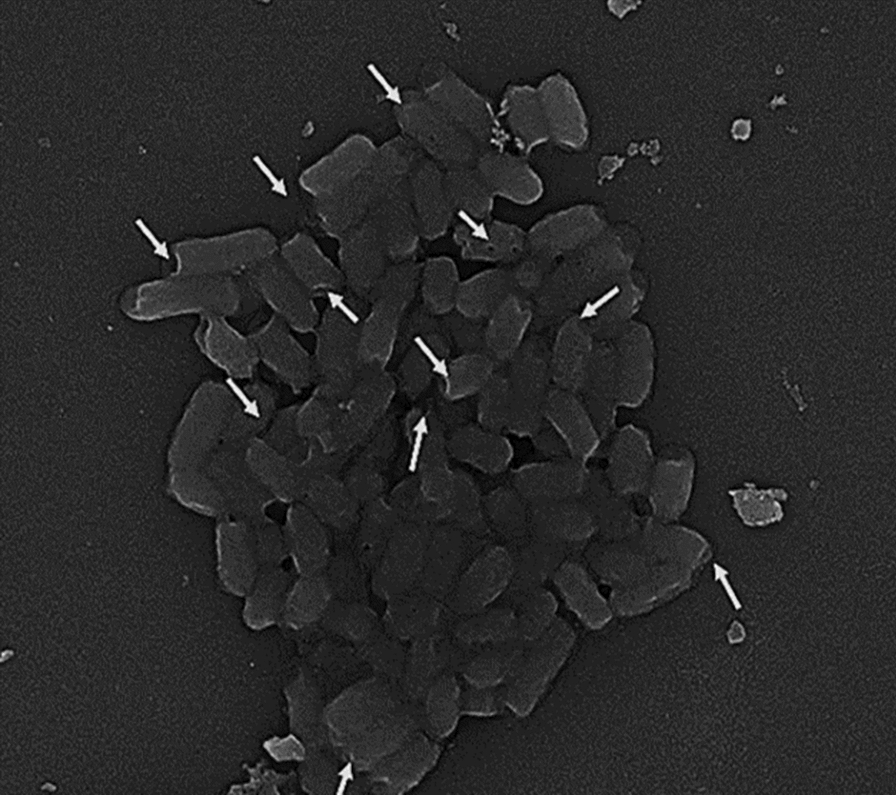
SEM image of *Sf*-BGCs/TX100. Scanning electron microscope (SEM) image of *Sf*-BGCs/TX100 pellet at magnification power × 16,000. White arrows point to a transmembrane tunnel formed in the cell walls of the cells treated with (5%) v/v TX100 for 12 h

Intact cell walls and several transmembrane tunnels were visible in *Sf*-BGC/TW80 and *Sf*-BGC/TX100, indicating efficient BGC preparation as shown in Figs. 2 and 3, respectively.

#### In vivo quality check “Stool cultures”

**Fig. 4:**
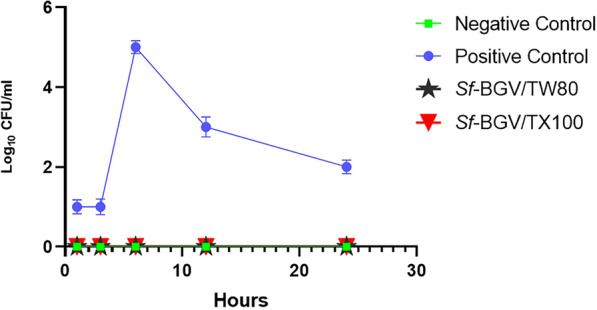
Viable counts of stool samples obtained from all tested groups of immunization and challenge experiment at different time intervals. A graph representing viable CFUs of *S. flexneri* 2b serotype (ATCC 12022) cultivated from stool samples collected from all tested groups (negative control “non-immunized non-challenged”, positive control “non-immunized challenged with ATCC 12022”, *Sf-*BGV/TW80 “immunized with *S. flexneri* bacterial ghost vaccine treated with (TW80) then challenged with ATCC 12022”*,* and *Sf*-BGV/TX100 “immunized with *S. flexneri* bacterial ghost vaccine treated with (TX100) then challenged with ATCC 12022″). The X-axis represents four different time slots at which the stool samples were collected. The Y-axis represents the Log_10_CFU/mL of ATCC 12022. Data represent mean ± standard errors of the mean (SEM). Significant difference between both treatments obtained using Two-way ANOVA data analysis, and considered significant if p < 0.0001, statistical analysis obtained using GraphPad Prism (8.0.2)

Stool samples taken prior to and following each dose of *Sf*-BGV/TX100 and *Sf*-BGV/TW80 ensured the safety of the BGVs by revealing no *S. flexneri* colonies. In addition, no *S. flexneri* was found in the stools of mice immunized with BGVs from either group after being challenged with *S. flexneri* 2b serotype ATCC 12022. Also, throughout the entire experiment, the negative control group failed to produce any viable CFUs. The non-immunized challenged group (positive control) from the pilot study and the immunogenicity evaluation of BGVs gave viable CFUs of *S. flexneri* in all mice at 3 h post-challenge and yielded the highest viable CFUs at 6 h post-challenge (1 × 10^5^ CFU/mL). Worth to mention that, colonies began to decline after 24 h of challenge, Fig. 4. All animal groups from the pilot study and immunogenicity evaluation of BGVs showed a (100%) survival.

### Immunization and challenge experiment “faecal pathology”

Pathological scoring of stool samples collected from all immunized groups with the BGVs weather before or after immunization and after challenge with *S. flexneri* 2b serotype ATCC 12022 gave normal stool features (brown, normal consistency, and absence of diarrheal episodes); the negative control group also gave the same consistent results throughout the whole experiment. Contrarily, the non-immunized challenged group with *S. flexneri* 2b serotype ATCC 12022 showed abnormal pathological features of stool post-challenge. The stool features started to change after 3–24 h post-challenge with ATCC 12022, as shown in Additional file 1: Figure S1.

### Bacteriological analysis of colon tissues

Homogenized colon tissues from immunized challenged groups with BGVs and the negative control group produced no viable CFUs of *S. flexneri*. In contrast, the positive control group (non-immunized challenged with *S. flexneri* 2b serotype ATCC 12022) gave average viable CFUs of (2 × 10^4^ CFU/mL).

### Determination of serum bactericidal activity assay (SBA) of “*Sf*-BGVs”

**Fig. 5:**
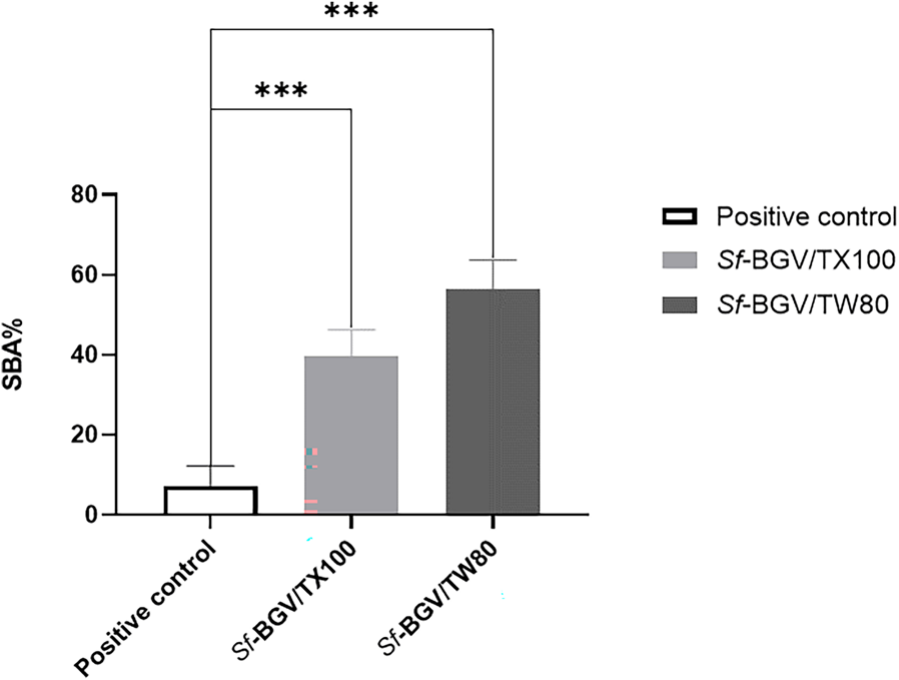
Serum bactericidal activity assay (SBA) of immunized and non-immunized challenged groups. A bar chart representing the serum bactericidal activity assay "SBA" calculated for all groups after 2 weeks of the last immunization dose The X-axis represents the tested groups (negative control “non-immunized non-challenged”, positive control “non-immunized challenged with *S. flexneri* 2b serotype ATCC 12022”, *Sf-*BGV/TW80 “immunized with *S. flexneri* bacterial ghost vaccine treated with (TW80) then challenged with *S. flexneri* 2b serotype ATCC 12022”*,* and *Sf*-BGV/TX100 “immunized with *S. flexneri* bacterial ghost vaccine treated with (TX100) then challenged with *S. flexneri* 2b serotype ATCC 12022”). The Y-axis represents the recorded SBA% for each group. (***) indicates a significant difference (P < 0.0001) compared to the positive control. The mean and standard deviation were obtained using one-way ANOVA data analysis

The average number of colonies found on (negative control/PBS) plates was (203). The bactericidal activities of the sera from the BGV-immunized groups increased significantly (P < 0.0001) in both groups. *Sf*-BGV/TX100 and *Sf*-BGV/TW80 had SBA rates of (40%) and (56%), respectively, whereas the positive control (non-immunized challenged) group only had an SBA of (7%), Fig. 5.

### Specific humoral immune response (IgG)

**Fig. 6:**
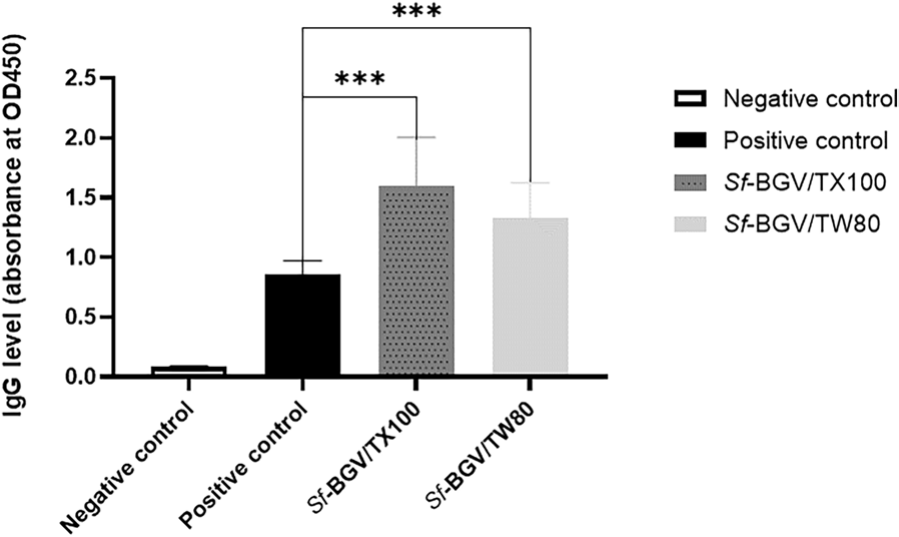
Serum specific antibody response (IgG) of immunized and non-immunized challenged groups. A bar chart representing specific antibody responses (IgG) measured by indirect ELISA for all tested groups The X-axis represents the tested groups (negative control "non-immunized non-challenged", positive control "non-immunized challenged with *S. flexneri* 2b serotype ATCC 12022", *Sf-*BGV/TW80 "immunized with *S. flexneri* bacterial ghost vaccine treated with (TW80) then challenged with *S. flexneri* 2b serotype ATCC 12022"*,* and *Sf*-BGV/TX100 "immunized with *S. flexneri* bacterial ghost vaccine treated with (TX100) then challenged with *S. flexneri* 2b serotype ATCC 12022"). The Y-axis represents the recorded IgG level (absorbance at OD450) for each group. (***) denotes a significant difference between the tested groups (P < 0.0001). The data represent the mean ± standard errors of the mean obtained using one-way ANOVA data analysis by GraphPad Prism (8.0.2) software

The serum IgG levels in the *Sf*-BGV/TX100 and *Sf*-BGV/TW80 groups were four and three times greater than those in the non-immunized challenged (positive control) and non-immunized non-challenged (negative control) groups, respectively. The two BGVs' IgG serum levels were not significantly distinct from one another (P > 0.05), although there was a significant difference between the immunized challenged groups and the negative control (P < 0.0001), Fig. 6.

### Histopathology analysis of colon tissues

**Fig. 7:**
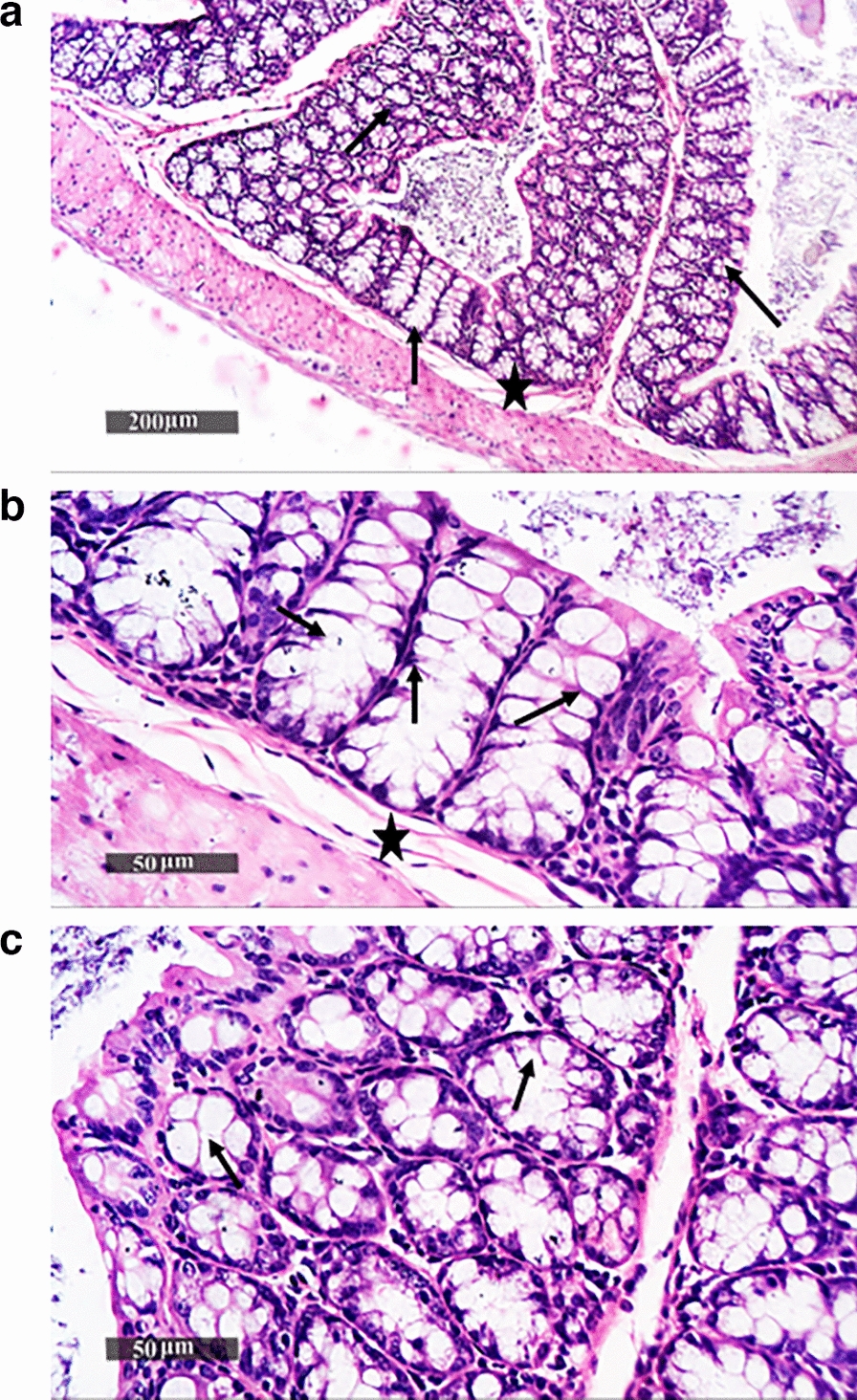
Histopathological image of colon tissues of the negative control (non-immunized non-challenged) group. (**a**, **b** and **c**) represent examination of tissues demonstrated well-organized histological features of the colonic wall, with intact intestinal crypts showing abundant records of goblet cells (arrow) alternated with many enterocytes with intact nuclear and subcellular details, intact propria, as well as submucosal layers (star) without abnormal infiltrates and normal vasculatures

**Fig. 8:**
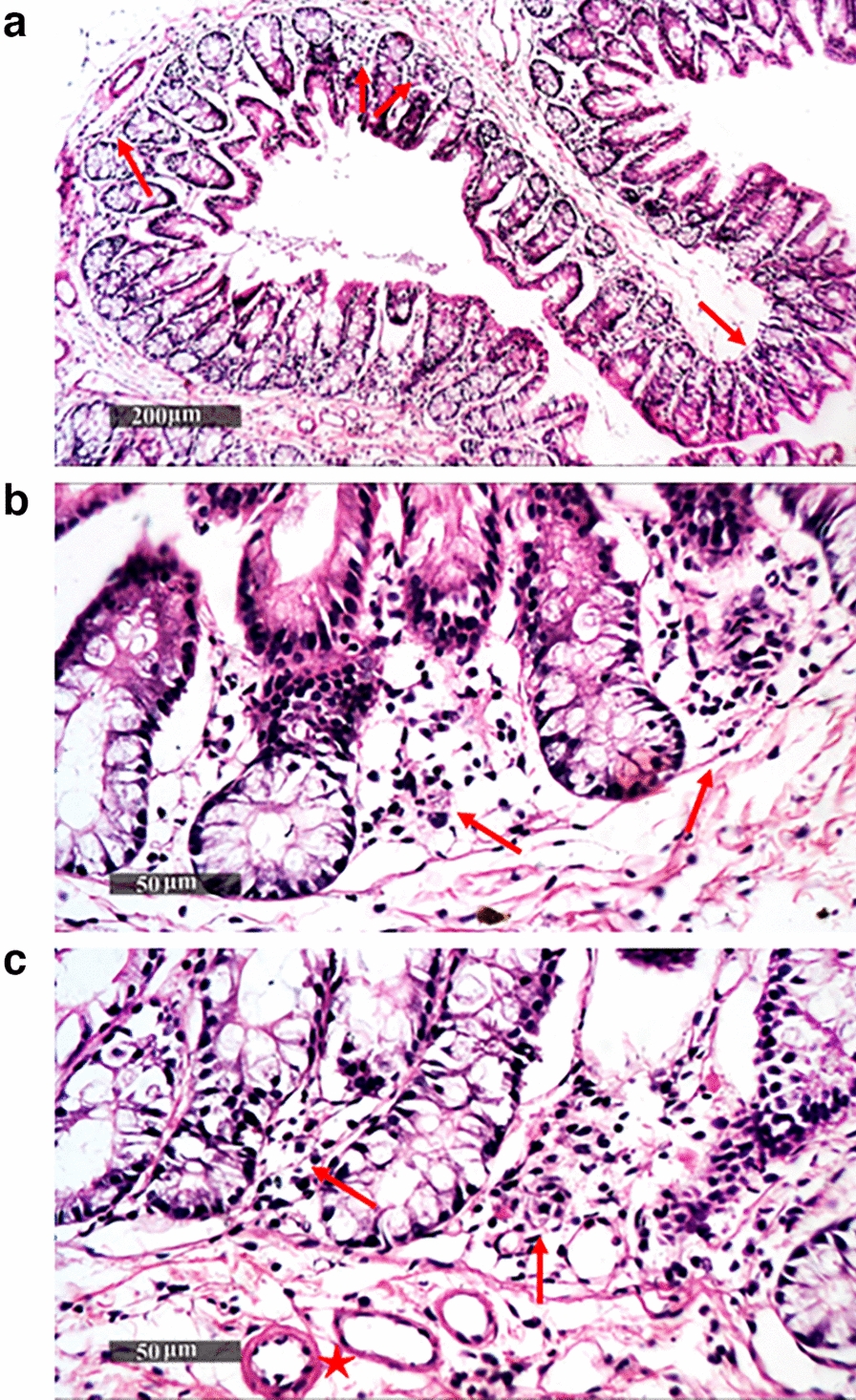
Histopathological image of colon tissues of the positive control group (non-immunized challenged with *S. flexneri* 2b serotype “ATCC 12022”). (**a**, **b** and **c**) represent examination of tissues showed moderate records of focal mononuclear inflammatory cells in the submucosal layer *(*red arrow*)* and between intestinal crypts with mild degenerative changes in lining epithelial cells, accompanied by moderate dilatation of submucosal blood vessels (red star*)*

**Fig. 9:**
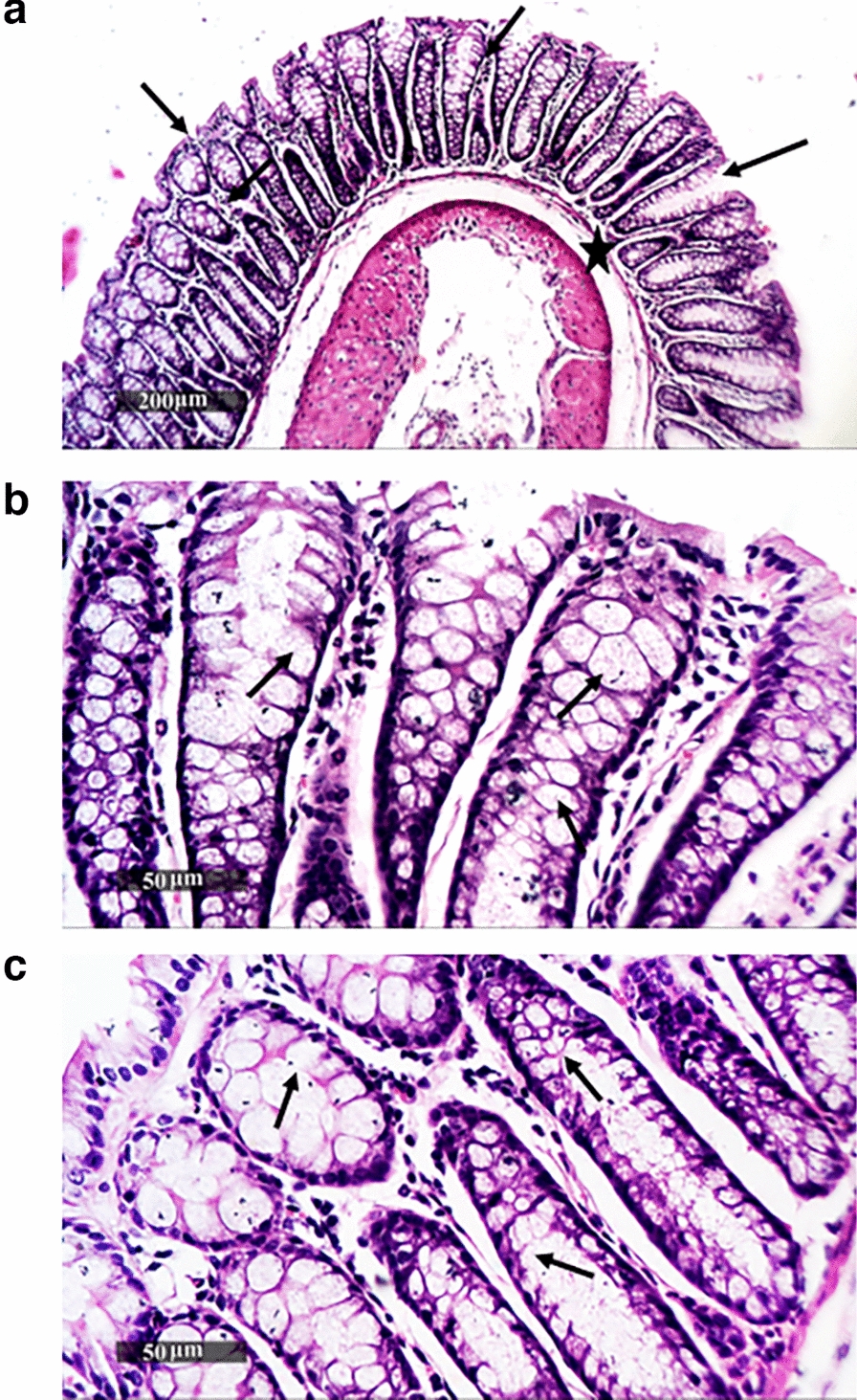
Histopathological image of colon tissues of *Sf*-BGV/TW80 (immunized with *S. flexneri* bacterial ghost vaccine produced by TW80 and challenged with *S. flexneri* 2b serotype “ATCC 12022”). (**a**, **b** and **c**) represent examination of tissues showed almost intact morphological features of the colonic wall in addition to integral submucosal layer (star) with intact glandular elements and minimal records of inflammatory cell infiltrates. Numerous goblet cell recordings were displayed in the intestinal crypts (arrows)

**Fig. 10:**
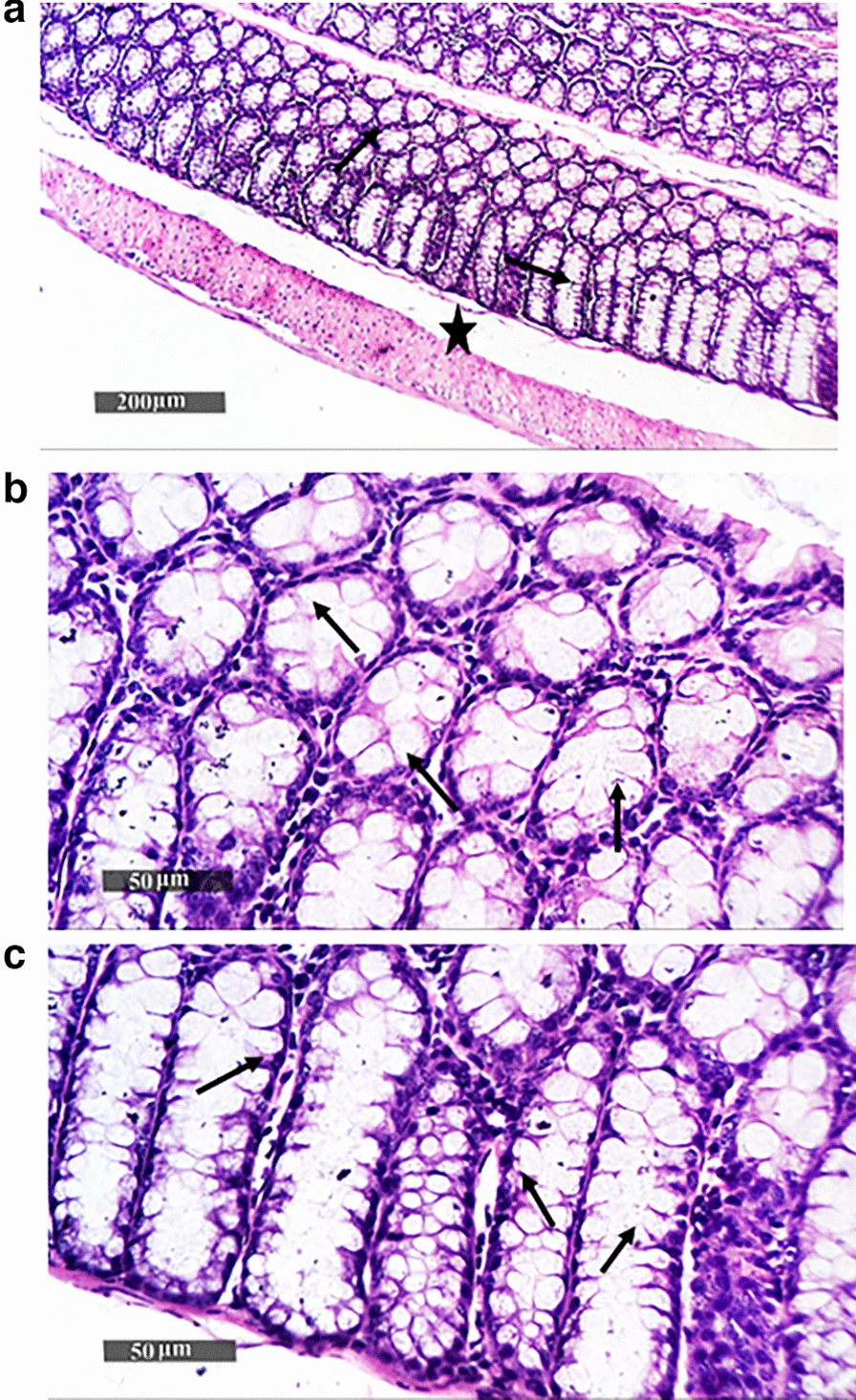
Histopathological image of colon tissues of *Sf*-BGV/TX100 (immunized with *S. flexneri* bacterial ghost vaccine produced by TX100 and challenged with *S. flexneri* 2b serotype “ATCC 12022”). (**a**, **b**, and **c**) demonstrate intact morphological features of the colonic wall, as well as an integral submucosal layer (star) with intact glandular elements and limited evidence of inflammatory cell infiltrates. Numerous goblet cell recordings (arrows) were seen in the intestinal crypts

Histopathological examination of colon tissue sections of the non-immunized, non-challenged (negative control) group showed well-organized histological features of the colonic wall, with intact intestinal crypts showing abundant records of goblet cells alternated with many enterocytes with intact nuclear and subcellular details, intact propria, as well as submucosal layers without abnormal infiltrates and normal vasculatures, Fig. 7. In contrast, the non-immunized challenged (positive control) group showed moderate records of focal mononuclear inflammatory cells in the submucosal layer and between intestinal crypts, with mild degenerative changes in lining epithelial cells, accompanied with moderate dilatation of submucosal blood vessels*.* Figure 8. Regarding *Sf*-BGV/TW80 and *Sf*-BGV/TX100, their colonic tissue sections showed almost intact morphological features of the colonic wall with intact glandular elements and minimal records of inflammatory cell infiltrates (resembling normal controls**),** Figs. 9 and 10, respectively. Results of the scoring system are shown in Additional file 1: Table S4.

## Discussion

BGCs are empty bacterial cells formed by transmembrane tunnel development within bacterial cell walls [20, 21]. One of the distinguishing features of BGCs that makes them intriguing for vaccine development is that they are the inactivated form of bacterial cells in which proliferation is impossible. As a result, BGCs are a significantly safer alternative to live vaccinations. The most alluring feature of BGCs is that they preserve the same cell membrane structure along with the antigenic proteins as live bacteria, with intact membrane protein piles and other immune-stimulating components such as lipids, polysaccharides, and peptidoglycans. Eventually, they were recognized by related membrane receptors of macrophages and dendritic cells, effectively phagocytosed, and triggered related immune effects such as T cell activation and mucosal inflammation. Furthermore, because BGCs combine immunogenicity and adjuvant action, they can be used as vaccines without the inclusion of additional adjuvants [22, 23]. However, because of the lack of chemical or physical inactivation during BGC formation, the majority of the important immunogenic determinants of the bacterial cell wall remain intact. Furthermore, all genetic material was removed from these cells, reducing the possibility of horizontal gene transfer [24]. When compared to inactivated or live vaccines, animals vaccinated with BGCs demonstrated a protective and favorable immunological response [16, 22, 24].

The main concept for producing successful batches of BGCs is to induce the formation of "transmembrane tunnels" on the bacterial cell wall. Our study targeted the non-ionic nature of the used surfactants (TX100 or TW80) to cause partial solubilization and disintegration of cell wall hydrophobic components. Furthermore, because of lactic acid's ability to enter the cytoplasmic membrane in its undissociated state, weak patches and small punctures known as "transmembrane tunnels" emerge, where the intracellular pH is decreased and the transmembrane proton motive force is interrupted [18, 25]. Previously, this approach was employed to generate Gram-negative BGCs of *Salmonella enterica serovar typhimurium* ATCC 13311 using (7%) v/v TW80 [18]. Using that protocol for BGC's production paves the way towards including new chemical treating agents with the same characteristics as TW80. Our study created a standardized technique for Sf- BGCs production by treating the cells with TX100 as a chemical inducer for BGC formation. TX100 was difficult to use as a chemical agent for BGC production since it is known for its damaging effect on bacterial cell walls and has a modulated corrosive action dependent on concentration and length of exposure [26]. As a result, finding the (MIC) of TX100 against ATCC 12022 was critical for making successful batches of BGCs, as was screening for the best incubation duration with the surfactants. To avoid the inhibitory impact of TX100 on ATCC 12022, the concentration employed to produce BGCs was less than the (MIC) value (sub-MIC) of (5%) v/v TX100 [18]. Although the sub-MIC concentration does not kill bacteria, it’s capable of influencing the physicochemical properties and architecture of the organism's outermost surface, as well as interfering with important bacterial cell functions such as adhesion and surface hydrophobicity [27]. Overall, employing that exact concentration strengthens the non-ionic nature and greatly aids in the formation of transmembrane tunnels. We discovered that incubation of ATCC 12022 with (5%) v/v TX100 for 24 h induced substantial cell damage, as seen under a light microscope. As a result, multiple incubation times were examined to determine the best, non-harmful incubation time. A successful batch of *Sf*-BGC/TX100 was produced by incubating ATCC 12022 with (5%) v/v TX100 for 12 h, which massively promotes the use of such a method as it requires a shorter preparation time in comparison to the TW80 method, which required 24 h of incubation with targeted cells. The successful production of these ghost cells was validated by intact cells that were visualized by light microscopy, the absence of growth upon recultivation, a significant amount of protein and DNA released compared to native untreated cells, and the presence of transmembrane tunnels on the cell walls confirmed by SEM [19].

Our findings slightly differed from previous study, when BGCs of *Salmonella enterica serovar typhimurium* ATCC13311 were effectively produced using TW80 (7%) at pH 3.6 [18]. In the case of *Sf*-BGC manufacturing, the pH was adjusted from 3.6 to 2.5 by adding 180 µL of lactic acid, only pH 2.8 and (5%) v/v TX100 or (7%) v/v TW80 were capable of creating BGCs. The large reduction in pH level was linked to the *Shigella* species' acid-tolerance ability, which facilitated their survival in those settings [28].

*S. flexneri* pathogenesis in humans begins with invasion and adhesion to the intestinal epithelial lining, facilitated by its virulence plasmid-encoded Type 3 Secretion System (T3SS), and continued to multiply in the target cell cytoplasm [29]. Furthermore, *Shigella's* pili play an important role in the overall bacterial-host pathogenesis [30]. *Shigella* species that cause dysentery are distributed worldwide, with *S. flexneri* being the most frequent in developing countries [31, 32]. It is worth mentioning that (90%) of patients with *S. flexneri* dysentery are serotypes 1b, 2a, 2b, 3a, and 6 [33]. In our investigation, we chose the 2b serotype ATCC 12022 strain as a BGC candidate and in the virulent challenge experiment. According to the reports, this strain colonizes the colonic region of the mice's guts in the same way as humans, causing massive changes to faecal pathology as observed in the non-immunized challenged group (positive control) [34]. That incident was confirmed by viable CFUs of *S. flexneri* in stool cultures and histopathological examination of non-immunized challenged with ATCC 12022, where intestinal inflammation and epithelial destruction occurred. Overall, that supports and mimics the pathogenesis of human shigellosis [35]. Notably, ghost cells must be tested for immunogenicity and safety before being considered as a potential vaccine candidate [36]. Since that male BALB/C mouse is susceptible to the *S. flexneri* 2a ATCC 12022 serotype and successfully infected by *Shigella* through oral route [34], we used the same mouse strain for the intraperitoneal pilot study to assess the progress of the infection and proceed with immunization and challenge experiment with *Sf*-BGVs. That pilot study was essential as it concluded that the infection lasted for 24 h and (100%) survival was recorded for all groups. Worth mentioning that, administrating ATCC 12022 inoculum as a virulent challenge using the (IP) route secured the successful infection, which was essential for evaluating the immunogenicity of BGVs [37]. This route expedites the fast and secure initiation of shigellosis; it directly passes through the peritoneal cavity, facilitating the invasion and attachment to colon tissues. This model of inducing shigellosis is considered useful for evaluating *Shigella* vaccine candidates and comprehending the bacillary dysentery induction mechanism caused by such infection, which came in line with our evaluation of the immunogenicity and safety of *Sf*-BGV/TW80 and *Sf*-BGV/TX100.

Until now, there has been no licensed vaccine for *Shigella* infection, despite the massive efforts that were directed toward this matter [8]. The primary objective in *Shigella* vaccine development is to produce a safe, effective, and economical vaccination to reduce mortality and morbidity due to dysentery and diarrhea caused by *Shigella* in low or middle-income countries ("LMICs"), travelers would also benefit from such a vaccine [38]. Additionally, using BGCs as a vaccine candidate is considered to be cheaper compared to other methods of *Shigella* vaccine development [8, 39]. All of the above-mentioned criteria happened to be fulfilled by the two batches of BGVs we’ve produced, as both of them induced a safe and specific immune response against *S. flexneri* 2b serotype ATCC 12022 after the IP challenge.

Superior results have been seen with *Sf-*BGV/TX100 and *Sf*-BGV/TW80 as they secured a strong and specific immune response (four and three-fold increase, respectively) compared to the non-immunized challenged group (positive control). The physical and chemical features of the antigen and host immune systems influence the strength of immune responses [40]. It is worth mentioning that pathogen-associated molecular pattern molecules (PAMPs) are found in Gram-negative bacterial shells that lack internal nucleic acids [10]. One of the most prominent PAMPs present on the surface of *Shigella* species is lipopolysaccharide (LPS), which can activate a significant immune response mediated by Toll-like receptor 4 (TLR4), its mostly expressed on the surfaces of antigen-presenting cells (APCs) [41, 42]. Above all, BGCs can boost host innate immune responses to antigens when used as vaccines or vaccine adjuvants. Successfully produced BGCs can activate APCs via the binding of LPS on BGCs to TLR4 in these cells [43, 44]. In line with the previously mentioned relationships between BGCs and the host immune system, it can be concluded that using such a method of producing *Sf*-BGCs can successfully induce a positive and safe immune response against shigellosis.

Moreover, both preparations demonstrated bacterial killing activities (significantly high SBA levels) without devastating effects on the colon tissues, as confirmed in histopathology examinations of immunized and challenged groups. Those BGVs also overcame the challenge for whole-cell vaccine developers as they struck a balance between reactogenicity and immunogenicity in *Shigella* vaccine development. In our murine model, we didn’t observe any of the adverse effects of reactogenicity (clinical symptoms or damaged colon tissues in histopathology analysis) after the administration of BGVs throughout the immunization period. Moreover, since *Sf-*BGV/TX100 is approved to be more immunogenic and quicker to produce than *Sf*-BGV/TW80, a complex multivalent universal *Sf-*BGV/TX100 could be designed by combining the 19 serotypes of *S. flexneri,* which include 1a, 1b, 1d, 2a, 2b, 3a, 3b, 4a, 4av, 4b, 5a, 5b, 6, X, Y, Xv, Yv, 7a, and 7b [33, 45, 46]. If the above-mentioned universal vaccine against *S. flexneri* is made, a safety check should be carried out in vitro and in vivo before any pre-clinical or clinical trials for such preparation.

## Conclusion

This study succeeded in creating effective, and safe *S. flexneri* BGV with a new standardized technique in the field of BGCs preparation. *Sf*-BGV/TX100 elicited a strong and specific immune response against *S. flexneri* ATCC 12022 in BALB/C mice. Histopathological studies confirmed and assured that both formulations were safe and owned no detrimental effect on mice. These findings pave the way for additional research regarding reactogenicity and the absolute safety of BGVs in pre-clinical and clinical trials.

## Methods

### Bacterial strain and culture conditions

KWIK-STIK of *S. flexneri* 2b serotype ATCC 12022 (Microbiologics, USA) was used in this study. For dissolving the bacterial strain, each stik was punched to release the hydrating fluid and mixed with lyophilized bacteria. ATCC 12022 was cultivated by the streak plate technique on deoxycholate citrate (DCA) (Oxoid, UK) agar under aerobic conditions at 37 °C for 24–48 h [47]. (30%) Luria–Bertani (LB) (Lab M, Neogen company, UK*)* glycerol stock was prepared for storing isolated colonies of *S. flexneri* 2b serotype ATCC 12022 at -80 °C for further use. *S. flexneri* 2b serotype ATCC 12022 was used for producing BGCs, as a control for protein and DNA measurements, and as a bacterial inoculum in the challenge experiment.

### Minimum inhibitory concentration (MIC) determination of “TX100” against ATCC 12022

Following the recommendations of the American Society for Microbiology, (TX100) (ADVENT, CHEMBIO PVT.LTD) was tested for its (MIC) against *S. flexneri* 2b serotype ATCC 12022 using the broth dilution method [48]. In short, *S. flexneri* 2b serotype ATCC 12022 inoculum was cultivated overnight in 5 mL LB at 37 °C, as 150 µL was transferred to 15 mL of fresh LB broth and incubated at 37 °C in a shaker incubator at 200 rpm for 3 h, the bacterial inoculum was adjusted at (1 × 10^6^ CFU/mL, OD_600_:0.2). Different dilutions of 10 mL of v/v TX100 were prepared (1, 2, 3, 4, 5, 6, 7, 8, 9, and 10%), and 100 µL of bacterial inoculum was added and incubated at 37 °C for 24 h. At last, a turbidity inspection was conducted in means of optical density (OD) determination at 600 nm for all incubated tubes, and the MIC value was recorded. The lowest TX100 concentration that showed no visible turbidity (complete bacterial killing) was recorded as the MIC value, and sub-MIC concentrations directly below the MIC value were used for BGC production. The sub-MIC concentration was tested at different incubation periods (1, 3, 6, 12 & 24 h) to obtain the ideal non-damaging incubation time for producing BGCs [18, 49].

### Bacterial ghost cell (BGCs) production

In this study, two sets of BGCs were prepared through incubation with two different types of surfactants, namely (TW80) and (TX100), each of which was used to prepare an individual preparation.

#### (TW80) method for production of *S. flexneri* BGC (*Sf*-BGC/TW80)

*Shigella flexneri* bacterial ghost cells *(Sf*-BGCs) were produced using the (TW80) method as described by Rabea et al*.* with some modifications [18]. A 100 µL of bacterial inoculum ATCC 12022 (1 × 10^10^ CFU/mL, OD_600_:0.6) was added to 10 mL of v/v (7%) TW80/LB (Alpha Chemika), and that mixture was incubated for 24 h at 37 ℃. After incubation, 180 µL of lactic acid was added to the treated cells in order to get the pH down to 2.8, and 1 h of incubation was performed. BGCs were centrifuged for 10 min at 4000 rpm, and the supernatant was saved for future use. The treated cells were washed and centrifuged twice at 4000 rpm with (0.45%) NaCl. Finally, BGC pellets were suspended in (0.45%) NaCl and kept at −20 °C for further use.

#### (TX100) method for production of *S. flexneri* BGC (*Sf*-BGC/TX100)

This BGCs set was prepared using 10 mL of v/v (5%) TX100/LB using the same bacterial inoculum (1 × 10^10^ CFU/mL, OD_600_:0.6). That concentration of (TX100) was corresponding to the sub-MIC value of surfactant against the bacterial strain ATCC 12022 (5%). The exact TW80 protocol was followed except for reducing the incubation time with the surfactant to 12 h only.

For each BGC's production method, biological and technical triplicates were performed.

### Quality check for the produced BGCs

#### In vitro quality check

The produced batches of BGCs went through a number of tests to ensure their quality in vitro. The presence of transmembrane tunnels in the cell wall of treated bacteria, negative growth upon re-cultivation, intactness, preservation of cellular integrity, and a substantial release of protein content and DNA from the treated cells in comparison to untreated native cells were assessed. All of the listed tests served as assurances of the successful production.

##### Light microscopic examination

For evaluating the cellular integrity and intactness of the treated cells, one loop from the preserved BGC pellets from each preparation was Gram-stained and visualized under the light microscope (× 1000).

##### BGCs re-cultivation

This step was performed to ensure that all living cells in the *S. flexneri* BGC pellets from each preparation had died during the manufacturing process. Samples from these pellets were sub-cultured for 24–48 h at 37 °C on DCA agar and LB broth. Colonies or turbidity (OD_600_) were observed, and any evidence of growth was noted.

##### Quantification of the released protein and DNA cellular contents

Using Nanodrop (Jenway-7415 NANO) at 280 nm and 260 nm, the released protein content and DNA were measured in the supernatant from the centrifuged BGCs, respectively [18, 50]. Quartz cuvette was used for both measurements, where an extinction *E*260 = 1 that corresponds to 50 μg dsDNAmL − 1 was used for DNA measurement. While for protein concentration measurements, Bovine Serum Albumin (BSA) standard curve was used [15]. Supernatants of centrifuged, native, untreated *S. flexneri* 2b serotype ATCC 12022 (1 × 10^10^ CFU/mL, OD_600_:0.6) cultured in LB for 12 h and 24 h at 37 ℃ served as controls for TX100 and TW80 preparations, respectively [50]. Three replicates of each BGC's preparation were used to measure the released protein and DNA contents. The average, standard deviation, and percentage of increase were computed.

##### Scanning electron microscopy (SEM) examination of “*Sf*-BGC”

Scanning electron microscopy (SEM Quanta FEG) was used to examine the morphological features (transmembrane tunnels) of the produced BGCs [16]. Cells were fixed for 2 h at room temperature in (2.5%) glutaraldehyde (SIGMA-Aldrich) in 0.1 M phosphate buffer (pH 7.0), washed three times with Phosphate-buffered saline (PBS), and then post-fixed for 1 h at room temperature in (1%) osmium tetroxide (SIGMA-Aldrich). For dehydrating the fixed cells, several grades of ethanol concentration (10, 30, 50, 70, and 100%) and liquid CO_2_ were used. Finally, after coating the cells with gold-palladium and mounting them on SEM stubs, they were examined using an SEM Quanta FEG with a 20 kV accelerating voltage and a x16000 amplification power. The identification of transmembrane tunnels on the surface of bacterial cells guaranteed the successful development of BGCs.

#### In vivo quality check “stool cultures”

Using an in vivo model, the viability of *Sf*-BGCs was also examined by culturing stool samples from experimental animals that had been immunized with the ghost cells on a selective medium (DCA). This step was conducted to ensure the safety of the used BGC preparation in the absence of living *S. flexneri*. Stool samples were taken before and after each dose of the bacterial ghost vaccines (BGVs), as well as before and after the *S. flexneri* 2b serotype ATCC 12022 (IP) challenge. Such samples were obtained using the procedures outlined in [51], with some modifications. Briefly, a fresh stool sample was collected by placing each mouse in a clean cage, where filter papers were placed underneath the mouse. A stool sample was collected using a sterile inoculating loop, suspended in 1 mL of sterile PBS, and vortexed for 1 min. Each sample was centrifuged for 1 min at maximum speed, and the supernatant was used for the viable count experiment. Briefly, 20 µL of each sample supernatant was serially diluted in 180 µL of sterile, cold PBS. Finally, 10 µL of each dilution was plated on DCA media and incubated for 24 h at 37 °C. Viable, colorless colonies of *S. flexneri* were counted, and CFU/mL was calculated for each plate using the following equation: CFU/mL=((No.of colonies x diltution factor))/(Volume plated)‎.

$${\text{CFU}}/{\text{mL}} = \frac{{({\text{No}}.\,{\text{of}}\,{\text{colonies}}\, \times \,{\text{diltution}}\,{\text{factor}})}}{{{\text{Volume}}\,{\text{plated}}}}$$

### Immunogenicity evaluation of BGVs

Animals were housed at a constant temperature of 25 °C in a 12 h light/ 12 h dark cycle with free access to regular pellet food and water. The mice were provided with a week of accommodations before the evaluation began. Before starting the immunogenicity evaluation experiment, a pilot study of (IP) infection with ATCC 12022 was carried out to ensure the successful infection in BALB/C mice. Six adult male BALB/C mice (6 weeks old at the day of the experiment) were divided equally into two groups, three in each, as follows: negative control (non-immunized received 20 µL of sterile PBS (IP), positive control (non-immunized infected once with *S. flexneri* 2b serotype ATCC 12022 (IP)). The progress of the infection was assessed through post-infection stool cultures in addition to monitoring the survival of the subjects for seven days. After a full assessment of the intraperitoneal infection in mouse subjects, the immunogenicity evaluation of BGVs began. Twenty-eight adult male BALB/C mice (6 weeks old on the day of the experiment) were divided equally into four groups, seven in each, as follows: negative control (non-immunized received sterile PBS), positive control (non-immunized infected with *S. flexneri* 2b serotype ATCC 12022), *Sf*- BGV/TW80 (immunized with BGC prepared with TW80 then challenged with *S. flexneri* 2b serotype ATCC 12022), and *Sf*-BGV/TX100 (immunized with BGC prepared with TX100 then challenged with *S. flexneri* 2b serotype ATCC 12022). Mice were first immunized with the BGVs to test their safety, and then they were challenged with the (IP) infection of *S. flexneri* 2b serotype ATCC 12022.

#### Immunization with BGVs

The (IP) route of immunization was used three times at intervals of 2 weeks [52, 53]. The TW80 and TX100 BGV groups received 20 µL (1 × 10^8^cells/mL) from each ghost preparation, while the negative control group received 20 µL of sterile PBS. To verify the lack of living *S. flexneri* cells and determine the safety of each preparation, stool samples were obtained from each mouse and plated on DCA media for counting viable CFU at 1, 3, 6, 12, and 24 h post-immunization with BGVs. Also, pathological scores of stool samples were assessed after each of the immunization doses of BGVs (1, 3, 6, 12, and 24 h post-immunization) as described in [53], color (brown, yellow, light green, blue-green), consistency (normal, loose, soft, hard), and an accumulative number of diarrheal episodes during the day (0–3).

#### Challenge experiment

The pure bacterial inoculum of *S. flexneri* 2b serotype ATCC 12022 was adjusted to (1 × 10^8^ CFU/dose) at OD_600_:0.4 for the challenge experiment, which began 2 weeks after the last immunization dosage (week 7). The actual bacterial inoculum was plated by serial dilution on DCA media prior to dose administration to confirm the infection and incubated for 24 h at 37 ℃. All groups received (IP) challenge dosage of *S*. *flexneri* 2b serotype ATCC 12022 20 µL once, except for the negative control, which received 20 µL of sterile PBS. Pathological scores of shigellosis were observed in all tested groups, as described in [53]. Also, stool samples were collected from all groups (1, 3, 6, 12, and 24 h post-challenge), plated on DCA media, and viable CFUs were counted.

### Blood collection and serum harvesting

After the completion of the immunization and challenge experiment (24 h post-challenge) for all groups under anesthesia, the retro-orbital sinus puncture blood collection method was carried out and termination procedures as described in [54, 55]. Sera were extracted from blood samples after coagulation (20–30 min) and centrifugation at 6000 rpm for 10 min. All samples were kept in sterile 1 mL microcentrifuge tubes and stored at −20 °C for further use.

### Bacteriological analysis

According to Vinod and his colleagues, the protective efficacy and safety of *Sf*-BGV preparations were evaluated against the virulently challenged group (positive control) [16]. At 24 h post-challenge, all of the experimental mice were sacrificed. Since the intestinal infection (shigellosis) was the focus of the immunization and challenge experiment, 0.25 cm of the colon was aseptically collected and intestinal content was evacuated and flashed with sterile, cold PBS. The collected tissues were manually homogenized in 1 mL of cold, sterile PBS. After being homogenized, all of the tissues were serially diluted ten times and then placed on DCA agar for 24 h at 37 °C. In terms of CFU/mL, the bacterial count for each culture plate was calculated.

### Determination of serum bactericidal activity assay (SBA) of “*Sf*-BGV”

Serum sample from animal groups were collected at the end of immunization and challenge experiment (24 h post-challenge). The procedure for determining serum bactericidal activity (SBA) is outlined in [16, 56, 57]. In short, 100 µL of *S. flexneri* 2b serotype ATCC 12022 adjusted at (1 × 10^6^ CFU/mL, OD_600_:0.2) and added to 25 µL of sera from immunized and non-immunized challenged groups. After incubating for 1 h at 37 °C, samples were cultured on DCA agar for 24 h at 37 °C. Serum was replaced with 25 µL of sterile PBS in the PBS/control tube. Using the following equation, the SBA% was determined:

$${\text{SBA}}\% \, = \,1\, - \,\frac{{\left( {{\text{number}}\,{\text{of}}\,{\text{viable}}\,{\text{bacteria}}\,{\text{after}}\,{\text{serum}}\,{\text{treatment}}} \right)}}{{\left( {{\text{number}}\,{\text{of}}\,{\text{viable}}\,{\text{bacteria}}\,{\text{after}}\,{\text{PBS}}\,{\text{treatment}}} \right)}}\, \times \,100\%$$

### Detection of specific humoral immune response (IgG) by Enzyme-Linked Immunosorbent assay (ELISA)

At the end of the immunization and challenge experiment (24 h post-challenge), all of the mice's sera were examined to determine the humoral immune response (IgG). A volume of 50 µL (1 × 10^9^ CFU/mL≈ OD_600_:0.5–0.6) of *S. flexneri* ATCC 12022 was used for coating 96-well microtiter ELISA plates for serum samples obtained from *Sf*-BGV/TW80, *Sf*-BGV/TX100, and the positive control group. The bacterial harvest was prepared by overnight culturing the bacterial strain in LB. The bacterial load was collected by centrifugation at 12,000 rpm for 5 min and the bacterial precipitates were sonicated in an ice bath for 5 min to break down the cells. The sonicated cells were centrifuged as previously mentioned, and the supernatants were collected. The protein content of cell lysates was determined using the Lawry method and was used to coat the ELISA plates using 1 µg/mL [58]. On the other hand, wells used for negative control samples were coated with irrelevant polypeptide obtained from *Streptococcus pyogenes* ATCC 19615 (Microbiologics, USA) that was cultivated in Tryptic Soy Broth (TSB) (Oxoid, UK) in order to test antibody specificity as a "mock vaccine”, where the same method of the coating was used [19, 22]. All plates were blocked using (1%) blocking buffer of bovine serum albumin (BSA) (SIGMA-Aldrich) for 1 h. Sera samples were twofold serially diluted and applied at 0.1 mL/well for 1 h at 37 °C. Serum dilutions were decanted, and plates were washed out using 200 µL of wash buffer. Goat anti-mouse IgG HRP per-oxidase conjugate (0.5 mg/mL) (Elabscience Biotechnology Inc.) was used as 0.1 mL of a 1/1000 final dilution. Plates were incubated for 1 h and washed as previously. All plates were treated with 100 µL of 3,3′,5,5′-Tetramethylbenzidine (TMB) substrate (Elabscience Biotechnology Inc.), and the produced color was stopped with 2N H_2_SO_4_. All experimental groups' mean optical densities were measured using microplate reader at 450 nm. To obtain the exact titer endpoint, sera were diluted twofold, and the highest dilution that gave an absorbance greater than three times that of the wells that received all treatments except the sera (the negative control) was used in the calculations.

### Histopathology analysis of colon tissues

From each of the tested groups, samples of the colon's tissue were taken. After flushing and being fixed for 72 h in (10%) neutral buffered formalin (Carolina Biological Supply Co.) samples were trimmed, processed in serial grades of ethanol, cleared in xylene, and infiltrated and embedded in Paraplast wax tissue embedding media (SIGMA-Aldrich). A rotatory microtome was used to cut 4-micron (µ)-thick sections of tissue that were mounted on glass slides from various samples. A Full HD microscopic imaging system (Leica Microsystems GmbH, Germany) was used to examine tissue sections after they had been stained with Hematoxylin and Eosin as a typical general morphological examination stain. The scoring system for lesions was used as described in [59]; the scoring system was: "Nil", as there were no visible lesions; + A mild lesion was found in less than (15%) of the tissue that was examined; ++A moderate lesion was found in (16–35%) of the tissue sections examined; +++ A severe lesion was found in more than (35%) of the tissue that was examined. Damage to the lining epithelium, infiltrates of inflammatory cells, and the condition of the blood vessels (congested or dilated) were the goals of the analysis. For sample fixation and staining, all standard procedures were followed [60].

### Statistical analysis

Statistical differences were analyzed using GraphPad Prism (8.0.2) (La Jolla, CA, USA) using one-way analysis of variance (ANOVA) and two-way analysis (ANOVA-mixed models); p-values with < 0.05 and 0.01 indicated significance and high significance, respectively.

### Supplementary Information

**Additional file 1: Table S1.** Minimum inhibitory concentration (MIC) determination of “TX100” against ATCC 12022. **Table S2. Sf**-BGC/TX100^*^ trials (initial parameters of the successful production of BGCs^**^). **Table S3.** *Sf*-BGC/TW80* trials (initial parameters of the successful production of BGCs**). **Table S4.** Scorning results from the histopathological examination of colon tissues. **Figure S1.** Fecal pathology of stool samples collected from positive control*, Sf*-BGV/TX100 and *Sf*-BGV/TW80 groups post-challenge with *S. flexneri* 2b serotype ATCC 12022*.* Stool samples collected from positive control (non-immunized challenged with *S. flexneri* serotype 2b ATCC 12022), *Sf*-BGV/TX100 (immunized with *S. flexneri* bacterial ghost vaccine treated with (TX100) then challenged with *S. flexneri* 2b serotype ATCC 12022) and *Sf*-BGV/TW80 (immunized with *S. flexneri* bacterial ghost vaccine treated with (TW80) then challenged with *S. flexneri* 2b serotype ATCC 12022) groups showed changes in the physical characters (color, consistency and presence of diarrheal episodes) collected at different time intervals post-challenge. The images showed changes in color, consistency and diarrheal episodes; which started to change post-challenge with ATCC 12022. (**a** and** b**) represent sample collected from *Sf*-BGV/TX100 and *Sf*-BGV/TW80 groups, respectively, at 12 h post-challenge with *S. flexneri* 2b serotype ATCC 12022. Those samples showed normal fecal pathology (normal brown color, intact stool and (0) diarrheal episode). (**c**, **d**, **e**, **f** and **g**) represent samples collected from positive control group post-challenge with *S. flexneri* serotype 2b ATCC 12022. **(c)** represents sample collected at (1 h) post-challenge with *S. flexneri* serotype 2b ATCC 12022; normal brown color, intact stool and (0) diarrheal episode. **(d)** represents sample collected at (3 h) post-challenge with *S. flexneri* serotype 2b ATCC 12022, turned to light brown, changes in consistency of the stool, (1–2) diarrheal episodes. **(e)** represents sample collected at (6 h) post-challenge with *S. flexneri* serotype 2b ATCC 12022, light brown, loss stool and (1–2) diarrheal episodes. **(f)** represents sample collected at (12 h) post-challenge with *S. flexneri* serotype 2b ATCC 12022, light brown color and continued diarrheal episode (> 2) in addition to presence of mucus. **(g)** represents sample collected at (24 h) post-challenged with *S. flexneri* serotype 2b ATCC 12022, stool turned to dark brown color with hard consistency and absence of diarrheal episode.

## Data Availability

All data obtained and analyzed during this study are included in this manuscript.

## Abbreviations

BGCs: Bacterial ghost cells
*S. flexneri*: *Shigella flexneri*
TX100: Triton X-100
TW80: Tween 80
BSA: Bovine serum albumin
OD: Optical density
SEM: Scanning electron microscopy
IP: Intraperitoneal
CFUs: Colony forming units
BGVs: Bacterial ghost vaccines
*S. sonnei*: *Shigella sonnei*
PMNs: Polymorphonuclear neutrophils
MIC: Minimum inhibitory concentrations
*Sf*-BGV: *Shigella flexneri* Bacterial ghost vaccine
*Sf*-BGC/TW80: *Shigella flexneri* Bacterial ghost cell produced by TW80
*Sf*-BGC/TX100: *Shigella flexneri* Bacterial ghost cell produced by TX100
*Sf*-BGV/TW80: *Shigella flexneri* Bacterial ghost vaccine produced by TW80
*Sf*-BGV/TX100: *Shigella flexneri* Bacterial ghost vaccine produced by TX100
LB broth: Luria–Bertani broth
DCA: Deoxycholate citrate agar
PBS: Phosphate buffer saline
T3SS: Type 3 Secretion system
TMB: 3,3′,5,5′-Tetramethylbenzidine substrate
PAMPs: Pathogen-associated molecular pattern molecules
LPS: Lipopolysaccharide
TLR4: Toll-like receptor 4
APCs: Antigen-presenting cells
