# Supplementary material for: Production of highly immunogenic and safe Triton X-100 produced bacterial ghost vaccine against Shigella flexneri 2b serotype
Source: Gut Pathog. 2023 Sep 7;15:41. doi: 10.1186/s13099-023-00568-7 (PMC10483756; doi:10.1186/s13099-023-00568-7)
Supplement: Supplementary file 1 — Additional file 1: Table S1. Minimum inhibitory concentration (MIC) determination of “TX100” against ATCC 12022. Table S2. Sf-BGC/TX100* trials (initial parameters of the successful production of BGCs**). Table S3. Sf-BGC/TW80* trials (initial parameters of the successful production of BGCs**). Table S4. Scorning results from the histopathological examination of colon tissues. Figure S1. Fecal pathology of stool samples collected from positive control, Sf-BGV/TX100 and Sf-BGV/TW80 groups post-challenge with S. flexneri 2b serotype ATCC 12022. Stool samples collected from positive control (non-immunized challenged with S. flexneri serotype 2b ATCC 12022), Sf-BGV/TX100 (immunized with S. flexneri bacterial ghost vaccine treated with (TX100) then challenged with S. flexneri 2b serotype ATCC 12022) and Sf-BGV/TW80 (immunized with S. flexneri bacterial ghost vaccine treated with (TW80) then challenged with S. flexneri 2b serotype ATCC 12022) groups showed changes in the physical characters (color, consistency and presence of diarrheal episodes) collected at different time intervals post-challenge. The images showed changes in color, consistency and diarrheal episodes; which started to change post-challenge with ATCC 12022. (a and b) represent sample collected from Sf-BGV/TX100 and Sf-BGV/TW80 groups, respectively, at 12 h post-challenge with S. flexneri 2b serotype ATCC 12022. Those samples showed normal fecal pathology (normal brown color, intact stool and (0) diarrheal episode). (c, d, e, f and g) represent samples collected from positive control group post-challenge with S. flexneri serotype 2b ATCC 12022. (c) represents sample collected at (1 h) post-challenge with S. flexneri serotype 2b ATCC 12022; normal brown color, intact stool and (0) diarrheal episode. (d) represents sample collected at (3 h) post-challenge with S. flexneri serotype 2b ATCC 12022, turned to light brown, changes in consistency of the stool, (1–2) diarrheal episodes. (e) represents sample collec [file 13099_2023_568_MOESM1_ESM.docx]

**Production of highly immunogenic and safe Triton X-100 produced bacterial ghost vaccine against *Shigella flexneri* 2b serotype.**

Amany Abdelfattah ^a,^ Reham Samir ^b*^ and Heba M. Amin ^a^.

**^a^** Department of Microbiology and Immunology, Faculty of Pharmacy, October University for Modern Sciences and Arts (MSA), Egypt, postal code (12451).

**^b^** Department of Microbiology and Immunology, Faculty of Pharmacy, Cairo University, Egypt, postal code (11562).

**Email addresses:**

Amany Abdelfattah ([aafattah@msa.edu.eg](mailto:aafattah@msa.edu.eg))

**^*^**Reham Samir ([reham.samer@pharma.cu.edu.eg](mailto:reham.samer@pharma.cu.edu.eg)) / **Corresponding author.**

Heba M. Amin ([htmagdy@msa.edu.eg](mailto:htmagdy@msa.edu.eg))

# **Supplementary data:**

## **1. Supplementary tables:**

| **Dilution** | **1%** | **2%** | **3%** | **4%** | **5%** | **6%** | **7%** | **8%** | **9%** | **10%** |
| --- | --- | --- | --- | --- | --- | --- | --- | --- | --- | --- |
| **Growth** | **+** | **+** | **+** | **+** | **+** | **+** | **-** | **-** | **-** | **-** |

**Table 1** Minimum inhibitory concentration (MIC) determination of “TX100” against ATCC 12022

Light blue shade = MIC value

| Incubation time with 5% v/v TX100 (hr.) | Growth (overnight culture) | The intactness of cells (Gram staining) |
| --- | --- | --- |
| 1 | **+** | **+** |
| 3 | **+** | **+** |
| 6 | **-** | **+** |
| 12 | **-** | **+** |
| 24 | **-** | **-** |

**Table. 2** Sf-BGC/TX100^*^ trials (initial parameters of the successful production of BGCs^**^).

**Note:** **Sf*-BGC/TX100 (*Shigella flexneri* Bacterial ghost cell treated with Triton X-100)

**BGCs (Bacterial Ghost Cells)

Light orange shade (optimum conditions for *Sf*-BGC/TX100 production)

**Table. 3** Sf-BGC/TW80* trials (initial parameters of the successful production of BGCs**).‎

| Incubation time with 7% v/v TW80 (hr.) | Growth (overnight culture) | The intactness of cells (Gram staining) |
| --- | --- | --- |
| 1 | **+** | **+** |
| 3 | **+** | **+** |
| 6 | **+** | **+** |
| 12 | **+** | **+** |
| 24 | **-** | **+** |

**Note:** **Sf*-BGC/TW80 (*Shigella flexneri* Bacterial ghost cell treated with Tween 80)

**BGCs (Bacterial Ghost Cells)

Light orange shade (optimum conditions for *Sf*-BGC/TW80 production)

**Table .4** Scorning results from the histopathological examination of colon tissues.

|  | Negative control | Positive control | *Sf*-BGV/TX100^*^ | *Sf*-BGV/TW80^**^ |
| --- | --- | --- | --- | --- |
| Lining epithelium damage | **-** | **++** | **-** | **-** |
| Inflammatory cells infiltrates | **-** | **++** | **-** | **-** |
| Congested/dilated blood vessels | **-** | **++** | **-** | **-** |

**Note:** **Sf*-BGV/TX100 (*Shigella flexneri* Bacterial ghost vaccine treated with Triton X-100)

***Sf*-BGV/TXTW80 (*Shigella flexneri* Bacterial ghost vaccine treated with Tween 80)

## **2. Supplementary figures:**


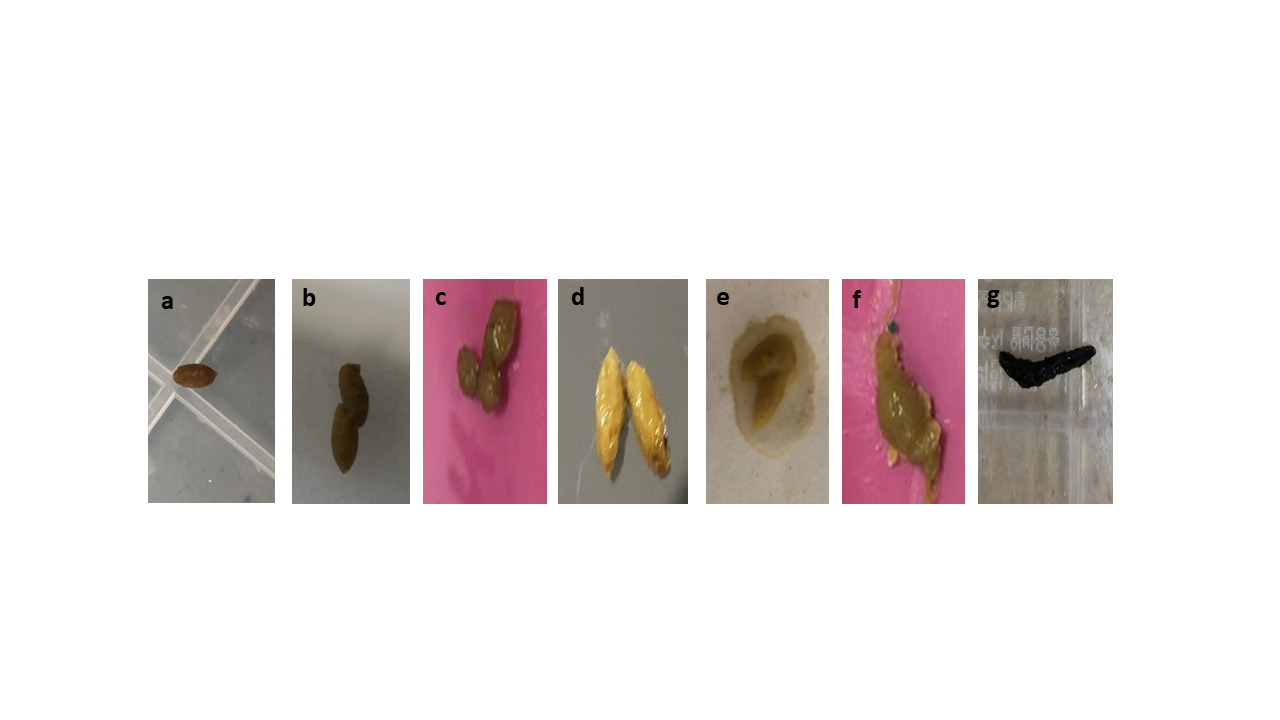


**Fig 1** Fecal pathology of stool samples collected from positive control, Sf-BGV/TX100 and Sf-BGV/TW80 groups post-challenge with S. flexneri 2b serotype ATCC 12022.
